# Altered galectin-3 distribution and migratory function in the pre-diabetic non-obese diabetic mouse thymus

**DOI:** 10.3389/fendo.2024.1200935

**Published:** 2024-10-17

**Authors:** Tiago Dutra Pereira Ramos, Ana Lucia Marques Ventura, Julia Pereira Lemos, Roger Chammas, Wilson Savino, Carla Eponina Carvalho-Pinto, Daniella Arêas Mendes-da-Cruz, Déa Maria Serra Villa-Verde

**Affiliations:** ^1^ Laboratory on Thymus Research, Oswaldo Cruz Institute, Oswaldo Cruz Foundation, Rio de Janeiro, RJ, Brazil; ^2^ National Institute of Science and Technology on Neuroimmunomodulation, Oswaldo Cruz Institute, Oswaldo Cruz Foundation, Rio de Janeiro, RJ, Brazil; ^3^ Laboratory of Neurochemistry, Department of Neurobiology, Biology Institute, Fluminense Federal University, Niterói, RJ, Brazil; ^4^ Center for Translational Research in Oncology, Instituto do Câncer do Estado de São Paulo, Hospital das Clínicas da Faculdade de Medicina da Universidade de São Paulo, São Paulo, SP, Brazil; ^5^ Comprehensive Center for Precision Oncology, Universidade de São Paulo, São Paulo, SP, Brazil; ^6^ Rio de Janeiro Research Network on Neuroinflammation, Oswaldo Cruz Institute, Oswaldo Cruz Foundation, Rio de Janeiro, RJ, Brazil; ^7^ INOVA-IOC Network on Neuroimmunomodulation, Oswaldo Cruz Institute, Oswaldo Cruz Foundation, Rio de Janeiro, RJ, Brazil; ^8^ Laboratory of Experimental Pathology, Department of Immunobiology, Biology Institute, Fluminense Federal University, Niterói, RJ, Brazil

**Keywords:** thymus, galectin-3, type 1 diabetes, NOD mice, extracellular matrix, thymocyte migration

## Abstract

Galectin-3 is an endogenous lectin which binds mainly to β-galactosides on the cell surface and extracellular matrix (ECM) glycoproteins. In the thymus, this lectin is constitutively expressed, being involved in thymocyte adhesion, migration, and death. Galectin-3 has been related to type 1 diabetes, an autoimmune disease characterized by pancreatic β-cell destruction mediated by autoreactive T lymphocytes. Non-obese diabetic (NOD) mice represent a suitable model to study type 1 diabetes, as they develop the disease like humans. We previously described important thymic alterations in these animals such as the development of giant perivascular spaces (PVS), characterized by the retention of T and B cells, intermingled with an ECM network, and associated with a defect in the expression of the fibronectin receptor VLA-5 and reduced sphingosine-1-phosphate receptor expression on developing thymocytes. In order to investigate galectin-3 expression in thymic microenvironmental cells and verify its interaction with cells and ECM molecules in PVS, we performed immunofluorescence following colocalization analysis in the thymic parenchyma of pre-diabetic NOD mice by confocal microscopy. In addition, thymocyte migration assays were performed to evaluate the effect of galectin-3 on NOD thymocyte migration. Herein, we showed a significant enhancement of colocalization with cortical and medullary thymic epithelial cells in NOD mice, as compared to controls. In the giant PVS of these animals, we observed a heterogeneous distribution of galectin-3, predominantly found in clusters of B lymphocytes and dendritic cells. Functionally, NOD thymocyte migratory response towards galectin-3 was impaired and a similar decrease was seen in transendothelial thymocyte migration. Taken together, our data provide the histological and functional background for a potential defective thymocyte migration involving galectin-3, thus placing this molecule as a further player in the intrathymic disturbances observed in pre-diabetic NOD mice.

## Introduction

The thymus is the primary lymphoid organ responsible for T cell differentiation. Histologically, it is divided into two well-defined regions: the cortex, where immature thymocytes are found, and the medulla, where mature thymocytes are lodged before leaving the organ (1, 2) Intermingled with developing thymocytes is the thymic microenvironment, mainly composed of non-lymphoid cells (mostly epithelial cells) and an extracellular matrix (ECM) network (3, 4). Particularly in the medulla, a third anatomical compartment is the perivascular space (PVS), located around blood vessels, being limited by an epithelial layer (5–7).

Thymic epithelial cells (TEC) modulate important intrathymic events, including T cell differentiation and migration, by direct cell-cell interactions and production of ECM proteins, cytokines, chemokines, hormones, and lectins (8–11). At the diverse stages of differentiation, thymocytes express specific receptors for cell-migration-related molecules. Accordingly, interactions between receptors and their corresponding ligands represent crucial molecular circuits that regulate thymocyte migration (12).

Galectin-3 (gal-3) belongs to a family of endogenous lectins that binds to β-galactoside residues present on the cell surface and on ECM glycoproteins (13). Gal-3 precipitates as a pentamer, facilitating interaction with multi-glycosylated proteins, with functional implications (14, 15). For example, gal-3 binds to integrins, including the fibronectin receptor VLA-5 (α5β1 integrin or CD49e/CD29), modulating integrin lateral mobility, cell adhesion and migration (16–18). In the thymus, gal-3 is predominantly expressed in the medulla and cortical-medullary junction. However, subcapsular, and cortical gal-3-positive cells are also found, including the epithelial component of the lymphoepithelial complex, the thymic nurse cells (19). A variety of thymic microenvironmental cells including TEC, macrophages, and dendritic cells (DC) express gal-3 (19, 20). We previously demonstrated the dose-dependent effect of gal-3 upon the interactions between thymocytes and thymic microenvironmental cells. At high concentrations, gal-3 promotes cell adhesion, whereas at low concentrations it inhibits TEC/thymocyte interactions, acting as a de-adhesive molecule (19). Among other intrathymic functions, exogenous gal-3 stimulates preferential migration of immature CD4^+^CD8^+^ thymocytes in the presence of laminin (21) and induces apoptosis of both CD4^-^CD8^-^ and CD4^+^CD8^+^ human thymocytes (22). Interestingly, we showed that the thymus of gal-3-deficient mice is reduced in size, presenting microenvironmental changes, decreased absolute thymocyte numbers and cell proliferation, and increased thymocyte apoptosis (23).

Recent reports have associated gal-3 with autoimmune diseases, including rheumatoid arthritis (24, 25), systemic lupus erythematosus (26), and type 1 diabetes (27, 28). The latter is an autoimmune disease characterized by the destruction of pancreatic β-cells; a process mediated mainly by autoreactive T cells (29). Data from the literature showed that gal-3 is involved in the development of type 1 diabetes by recruiting neutrophils and macrophages to pancreatic islets, leading to inflammation and β-cell death, which results in diabetes (reviewed in 30). The non-obese diabetic (NOD) mouse represents a suitable model to study type 1 diabetes since the animals develop the disease like humans (31). Several lines of evidence pointed out that the thymus plays an important role in NOD mouse autoimmunity (32–34) Previous studies revealed important abnormalities in the NOD mouse thymus, such as altered distribution of TEC, increased ECM deposition, and appearance of giant PVS. These anatomical compartments are filled with mature thymocytes and clusters of B cells, intermingled with an ECM network (35, 36). Due to the accumulation of lymphocytes in the giant PVS, we raised the hypothesis of impaired NOD thymocyte migration. In this respect, NOD thymocytes have decreased expression of VLA-5. This defect was further correlated to an abnormally diminished thymocyte migration driven by fibronectin, which could explain the retention of mature T cells in giant PVS (37).

We also showed that FoxP3^+^ regulatory T cells present a decrease in VLA-5 expression and are accumulated in giant PVS (38). These cells are important in the control of type 1 diabetes, as they prevent the beginning of immune response in pancreatic lymph nodes and subsequently in the pancreas (39). Furthermore, we recently observed a decreased expression of sphingosine-1-phosphate receptor 1 (S1P1) in the NOD mouse thymus in comparison with C57BL/6 controls, particularly in the mature single-positive CD4^+^CD62L^hi^ and CD8^+^CD62L^hi^ subpopulations, both being deficient in VLA-5 expression. Moreover, NOD CD62L^hi^CD49e^-^ thymocytes presented lower migratory response under different S1P doses, whereas CD62L^hi^CD49e^+^ subsets exhibited the opposite behavior. These results suggest that the modulation of S1P1 and S1P/S1P1 interactions also plays a role in the disturbances of thymocyte migration described in the NOD thymus (40).

Considering that gal-3 is a modulator of cell adhesion, migration, proliferation, and apoptosis and that it was previously related to the pathogenesis of type 1 diabetes, we investigated if gal-3 distribution in the NOD mouse thymus, especially in the giant PVS, could somehow interfere with the exit of mature thymocytes to the periphery, such as FoxP3^+^CD4^+^ T cells, favoring autoimmune reactions and diabetes. We also assessed whether the migratory response to gal-3 could be altered in NOD mouse thymocytes.

## Materials and methods

### Animals

Female BALB/c and pre-diabetic NOD mice aged 12-20 weeks or 4-5 weeks (when indicated) were obtained and maintained under specific-pathogen-free conditions at the animal facility of the Oswaldo Cruz Foundation (Rio de Janeiro, Brazil). BALB/c mice was used as a control group of the present study, because gal-3 expression was first standardized in cortical and medullary regions from thymus of this strain (19). In addition, BALB/c mice is considered a suitable control for study of thymic abnormalities of NOD mice (37). Unless stated specifically, at least 3 animals from each group were used in each experiment described below. All protocols for the use and care of animals were approved by the corresponding Ethics Committee for the Use of Experimental Animals (Oswaldo Cruz Institute, Rio de Janeiro), under license numbers L-024/09 and L-004/2014.

Evaluation of onset of diabetes in NOD animals was defined by weekly-based determination of glycemia, using the Prestige Smart System^®^ blood glucose test strips (Home Diagnostics, Fort Lauderdale, USA) following the manufacturer’s instructions. Mice with glycemia lower than 250 mg/dL were classified as pre-diabetic or non-diabetic (41).

### Antibodies and other reagents

The following primary antibodies were used: rabbit anti-gal-3 (sc-20157, Santa Cruz, Dallas, USA), anti-fibronectin (24951), anti-laminin (24851) from Novotec (Saint Martin-La-Garenne, France), anti-cytokeratin (Z0622, Dako, Carpinteria, USA), rat anti-CD4 (553647), anti-CD8 (553027), anti-CD19 (5502840) purchased from BD Pharmingen™ (San Jose, USA), anti-FoxP3 (14-5773-82, eBioscience, USA), F4/80 monoclonal antibody (ab6640, Abcam, Cambridge, USA), and biotinylated anti-CD11c (117303, Biolegend, San Diego, USA). Alexa Fluor 488-labeled goat anti-rat IgG (A11006), Alexa Fluor 633-labeled goat anti-rabbit IgG (A21070), and Alexa Fluor 488-labeled streptavidin (S11223), purchased from Molecular Probes (Carlsbad, USA), were used as second-step reagents. In triple staining with rabbit-derived primary antibodies, Zenon Alexa Fluor 488 (Z-25302) and Zenon Alexa Fluor 555 (Z-25305) rabbit IgG labeling reagents, both from Molecular Probes (Carlsbad, USA), were applied. Tissue-Tek O.C.T. (4583) was obtained from Sakura, Torrance, USA. Poly-L-lysine (P8920) and the aqueous mounting medium Fluoroshield (F6182) were purchased from Sigma-Aldrich (Saint Louis, USA).

### Immunofluorescence

BALB/c and NOD mouse thymuses (n=3/group) were removed, individually embedded in Tissue-Tek O.C.T., and frozen at -70°C. Five μm-thick cryostat sections were settled on poly-L-lysine-covered glass slides, fixed in ice-cold acetone for 10 min, and air-dried. Slides were washed with PBS (P4417, Sigma-Aldrich, Saint Louis, USA), followed by blocking with PBS-BSA 1% for 20 min. For CD4-gal-3-cytokeratin, CD8-gal-3-cytokeratin, CD19-gal-3-cytokeratin, F4/80-gal-3-cytokeratin triple staining, slides were incubated with rat primary antibody for 1h at room temperature (RT), washed with PBS three times for 5 min, incubated with Alexa Fluor 488-labeled goat anti-rat secondary antibody for 45 min at RT, washed with PBS three times, incubated with anti-gal-3 diluted in PBS-BSA 1%-saponin (S7900-25G, Sigma-Aldrich, Saint Louis, USA) 0.1% buffer overnight at 4°C, washed with PBS three times, incubated with Alexa Fluor 633-labeled goat anti-rabbit secondary antibody diluted in PBS-BSA 1%-saponin 0.1% buffer for 45 min at RT, washed with PBS three times, incubated with anti-cytokeratin antibody conjugated with Zenon Alexa Fluor 555 rabbit Ig labeling reagent for 2h and washed with PBS three times.

For CD11c-gal-3-cytokeratin staining, slides were first incubated with the biotinylated anti-CD11c antibody for 1h, washed with PBS three times, incubated with Alexa Fluor 488-labeled streptavidin for 45 min, washed with PBS three times and stained for gal-3 and cytokeratin. For FoxP3-gal-3-cytokeratin staining, slides were first incubated with anti-FoxP3 antibody diluted in PBS-BSA 1%-saponin 0.1% buffer for 1h, washed with PBS three times, incubated with Alexa Fluor 488-labeled labeled goat anti-rat secondary antibody diluted in PBS-BSA 1%-saponin 0.1% buffer for 45 min at RT, washed with PBS three times and stained for gal-3 and cytokeratin, as above described. For gal-3-fibronectin-cytokeratin and gal-3-laminin-cytokeratin staining, slides were stained for gal-3, incubated with anti-fibronectin or anti-laminin antibody conjugated with Zenon Alexa Fluor 488 rabbit Ig, washed, and incubated with anti-cytokeratin antibody conjugated with Zenon Alexa Fluor 555 rabbit Ig labeling reagent. All slides were ultimately mounted with Fluoroshield using coverslips. Negative controls were performed with unrelated IgG isotypes or incubating the cells with the secondary antibody alone. None controls yielded significant fluorescence signals.

The murine endothelioma cell line tEnd.1, kindly provided by Dr. Thereza Christina Barja-Fidalgo (University of the State of Rio de Janeiro, Brazil), was cultured in RPMI 1640 medium (R6504, Sigma-Aldrich, Saint Louis, USA) supplemented with 10% fetal calf serum (FCS 12657029, Invitrogen Life Technology, Waltham, USA), 2mM glutamine (G8540), 100U/mL streptomycin (S9137) (both from Sigma-Aldrich, Saint Louis, USA) in 8 well Permanox Lab-Tek^®^ chamber slides (177445, Thermo Scientific Nunc., Rochester, NY, USA) at 37°C in a CO_2_ humidified atmosphere. For gal-3 staining, cultures were fixed in methanol for 7 min, followed by blocking with PBS-BSA 1%, incubated with anti-gal-3 primary antibody for 1 h at RT, washed with PBS three times, incubated with Alexa Fluor 488-labeled goat anti-rabbit secondary antibody for 45 min at RT, washed with PBS three times. The slides were mounted with Fluoroshield using coverslips.

### Immunohistochemistry

Gal-3 expression was evaluated by immunohistochemistry on paraffin sections of thymus from BALB/c and pre-diabetic NOD mouse thymuses aged 4-5 weeks and 16 weeks. Initially, the sections were deparaffinized in three xylol baths for 3 min, hydrated in decreasing concentrations of alcohol for 3 min, washed in running water for 5 min and rinsed in distilled water. For antigen retrieval, sections were incubated with 3M urea (U5378-100G, Sigma-Aldrich, Saint Louis, USA) for 15 min. Next, the sections were washed with three baths of PBS-0.1% Tween 20 (P1379-25ML, Sigma-Aldrich, Saint Louis, USA) for 5 min. To block endogenous peroxidase, the sections were incubated with 3% H2O2 (386790-100ML, Sigma-Aldrich, Saint Louis, USA) diluted in 70% methanol for 5 min. After blocking, the sections were washed again with three baths of PBS-0.1% Tween 20 for 5 min. To block nonspecific binding, sections were incubated with 10% horse serum diluted in 2% PBS-BSA. After this incubation, the sections were incubated with anti-gal-3 for 30 min or overnight at 4°C in a humid chamber. After this incubation, the sections were washed in three baths of PBS-0.1% Tween 20 for 5 min. Next, the sections were incubated with anti-mouse secondary antibody conjugated to peroxidase for 1 h. After three consecutive washes with PBS-0.1% Tween 20, the reaction was revealed using the peroxidase substrate (H2O2) and 3,3-diaminobenzidine (DAB) (K3467, Dako, Glostrup, Denmark). After washing in running distilled water, the sections were counterstained with Harris Hematoxylin for 30 seconds, dehydrated in increasing concentrations of alcohol and clarified in xylene. The slides were mounted in Entellan ^®^ new (107961, Merck, Darmstadt, Germany). For quantitative analysis of gal-3 positive cells per area, ten random images of each animal’s thymus containing cortical or medullary regions were acquired under a 20x objective, using the Nikon Eclipse E600 microscope (Nikon Instruments Inc., Melville, NY, USA) with the DXM 1200F digital camera system (Nikon Instruments Inc., Melville, NY, USA). The occupied area was determined by a grid of dots and cells positive for gal-3 were counted using the Image J program (NIH). The result was represented by the number of gal-3-positive cells divided by the area occupied.

### Quantitative evaluation of fluorescence and colocalization analyses

Immunostained tissues were analyzed by confocal microscopy using a Leica TCS SP5 device (Leica Microsystems CMS GmbH, Mannheim, Germany) from the Confocal Microscopy Laboratory Station (Biology Institute, Fluminense Federal University, Niteroi, Rio de Janeiro). With respect to gal-3 quantitative fluorescence and colocalization analyses, at least six photomicrographs comprising cortical and medullary regions of the thymic lobules (n=3/group) were randomly obtained with 63x objective at zoom 2. Thymic cortical and medullary regions were defined by cytokeratin staining. Image J (NIH) and LAS AF Lite (Leica Microsystems) softwares were used to quantify fluorescence and colocalization, respectively. The result of gal-3 quantification was represented by fluorescence mean which is calculated by the sum of the gray values of all pixels divided by total pixel numbers. For gal-3 colocalization with CD4, CD8, CD19, fibronectin, and laminin in giant PVS, at least six photomicrographs comprising blood vessels containing cytokeratin-negative regions of NOD mouse thymus sections were selected. Before beginning the colocalization analysis, each photomicrography was adjusted through the establishment of percentage values for threshold and background parameters. A scatter diagram called a cytofluorogram was displayed for each photomicrography. This diagram shows the distribution of gray-scale values of fluorescent signals in two detection channels. A digital image was also generated with colocalizing fluorescence signals in white to facilitate visualizing the colocalization of two given molecules. The result was represented by the colocalization rate (%) which is calculated by the ratio of the area of colocalizing fluorescence signals by the area of the image foreground; the latter indicating the difference between the total area of the image and the area of the image background.

### Cell migration assays

To analyze the effect of gal-3 on thymocyte migration, we performed migration assays in 6.5 mm diameter transwell chambers bearing 5 µm pores (3421, Corning Inc., New York, USA) (19, 34). The membranes were previously treated with fibronectin from human plasma (F2006) or BSA (A7906) alone (10 μg/mL), both from Sigma-Aldrich (Saint Louis, USA), for 1h at 37°C, followed by blocking with PBS-BSA 0.5% for 45 minutes. For thymocyte isolation, thymuses from BALB/c and NOD mice were removed and mechanically macerated in a glass potter grinder with 1mL RPMI 1640. Next, thymocyte suspension were counted in a Neubauer chamber. In those membranes treated only with BSA we added 2.5x10^6^ thymocytes from both mice treated or not with recombinant mouse gal-3 (1197-GA, R&D Systems, Minneapolis, MN.) to the upper chamber (in 100 μL fugotaxis buffer containing 1% BSA in RPMI 1640 medium) and 600 μL of the same buffer in the lower chamber. After 3 hours of migration at 37°C in the CO_2_-humidified atmosphere, we washed the lower compartment of the membranes with 200μL of 1% RPMI 1640-BSA, collected the cells that migrated to the lower chamber, and counted them in a Neubauer chamber (21, 37).

To mimic thymocyte export under the influence of gal-3 to the periphery, we performed a reverse transendothelial migration assay (38), in which we used the murine thymic endothelium cell line t.End.1. These cells were plated on the lower compartment of Transwell^®^ system membranes with 8 µm pores (3422, Corning Inc., New York, USA) for 2 hours to allow adhesion. Then the cells were cultured in 500μL of RPMI 1640 and added to the upper and lower chambers for 48 hours at 37°C in a CO_2_-humidified atmosphere. After this incubation period, 2.5x10^6^ thymocytes from BALB/c or NOD mice, treated or not with recombinant gal-3, were placed in the upper chamber (in 100 μL of migration buffer) and 600 μL of the same buffer were added to the lower chamber. After overnight migration, thymocytes were collected in the lower chamber and counted in a Neubauer chamber.

### Statistical analyses

Data distribution was analyzed using the D’Agostine-Pearson normality test. Statistical differences were analyzed by unpaired Student’s *t-*test or one-way ANOVA, followed by *post hoc* Tukey’s test using GraphPad Prism 5 software (GraphPad^®^ Software, San Diego, USA). The results were expressed as mean ± standard error (SE). The differences between the analyzed groups were considered statistically significant when *p*<0.05.

## Results

### Increase of gal-3 deposition in the thymus of pre-diabetic NOD mice

**Figure 1:**
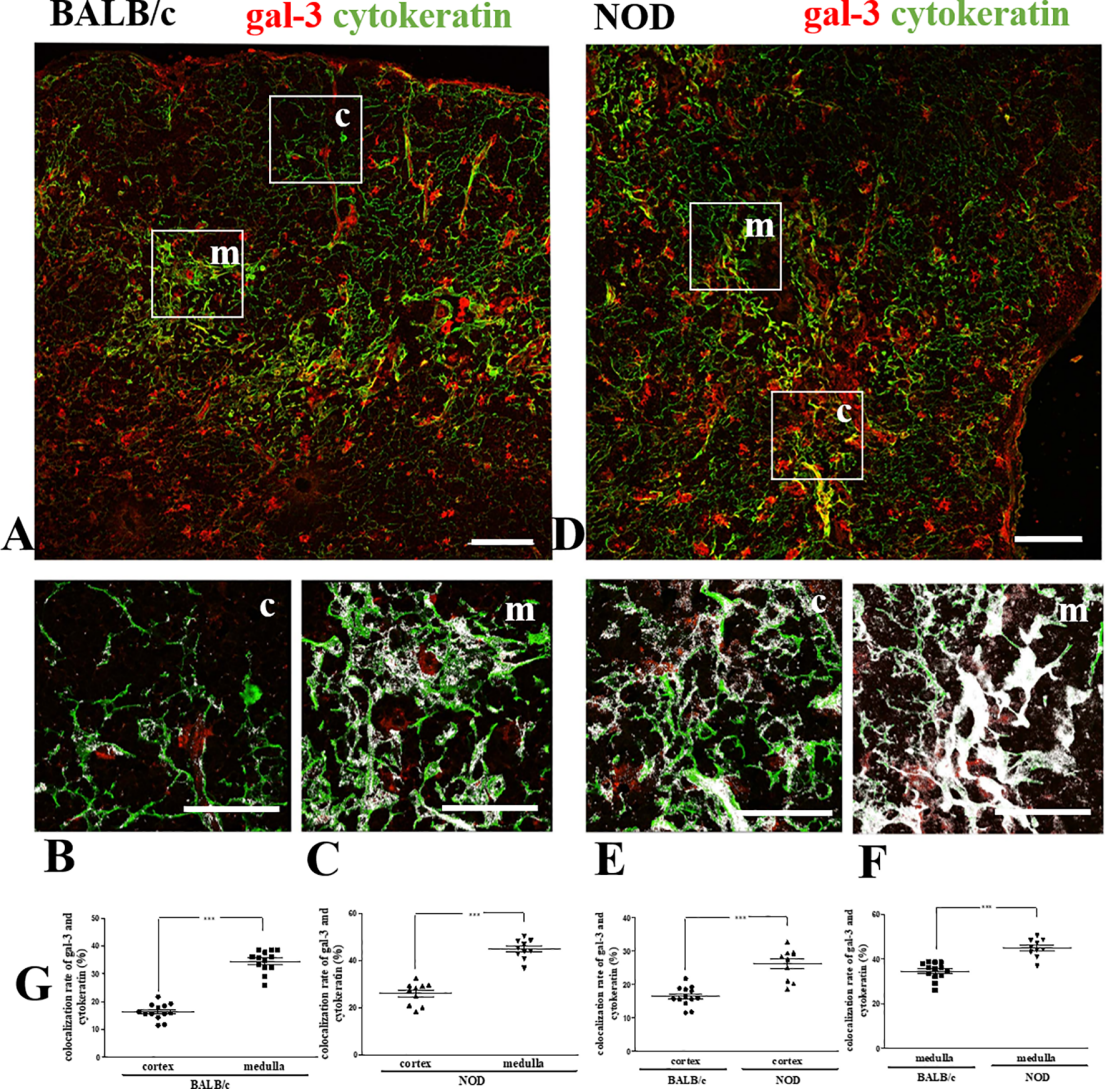
Colocalization of gal-3 with TEC in pre-diabetic NOD mouse thymus. Double immunostaining for gal-3 (red) and cytokeratin (green) in BALB/c **(A–C)** and pre-diabetic NOD mouse **(D–F)** thymus sections. Bar: 100 μm. Lower panels depict gal-3 labeling in the cortex and medulla respectively of BALB/c **(B, C)** and NOD **(E, F)** mouse thymic lobules, with white areas high-lightening the gal-3/cytokeratin colocalization. Bar: 50 μm. **(G)** Graphs show the quantitative analysis of colocalization rates of gal-3 and cytokeratin in cortical and medullary regions of BALB/c and pre-diabetic NOD mouse thymuses. Ten to thirteen photomicrographs per group were analyzed (n=3 mice/group). c- cortex; m- medulla. Data were analyzed by unpaired Student’s *t* test and expressed as mean± SEM. ***p< 0.001.

We initially investigated the expression of gal-3 in the thymus of pre-diabetic NOD mice. To study the *in situ* expression of gal-3 in TEC, we double-stained thymus sections of BALB/c (**Figures 1A–C**) and NOD (**Figures 1D–F**) for gal-3 and cytokeratin, followed by confocal microscopy analysis. Similar to what we had previously shown for BALB/c mice (19), we observed a stronger gal-3 immunostaining in medullary (**Figure 1F**) than in cortical regions (**Figure 1E**) of NOD mouse thymuses, including both epithelial and non-epithelial (cytokeratin-negative) cells. Quantitative analysis of the colocalization between gal-3 and cytokeratin in NOD mouse thymuses showed a significant enhancement of colocalization in the medulla, as compared to the cortex (**Figure 1G**). Importantly, we noticed a significant increase of colocalization in the cortex as well as in the medulla of NOD mouse thymuses in comparison to BALB/c, suggesting that NOD cortical and medullary TEC express more gal-3 than the corresponding BALB/c TEC (**Figure 1G**).

**Figure 2:**
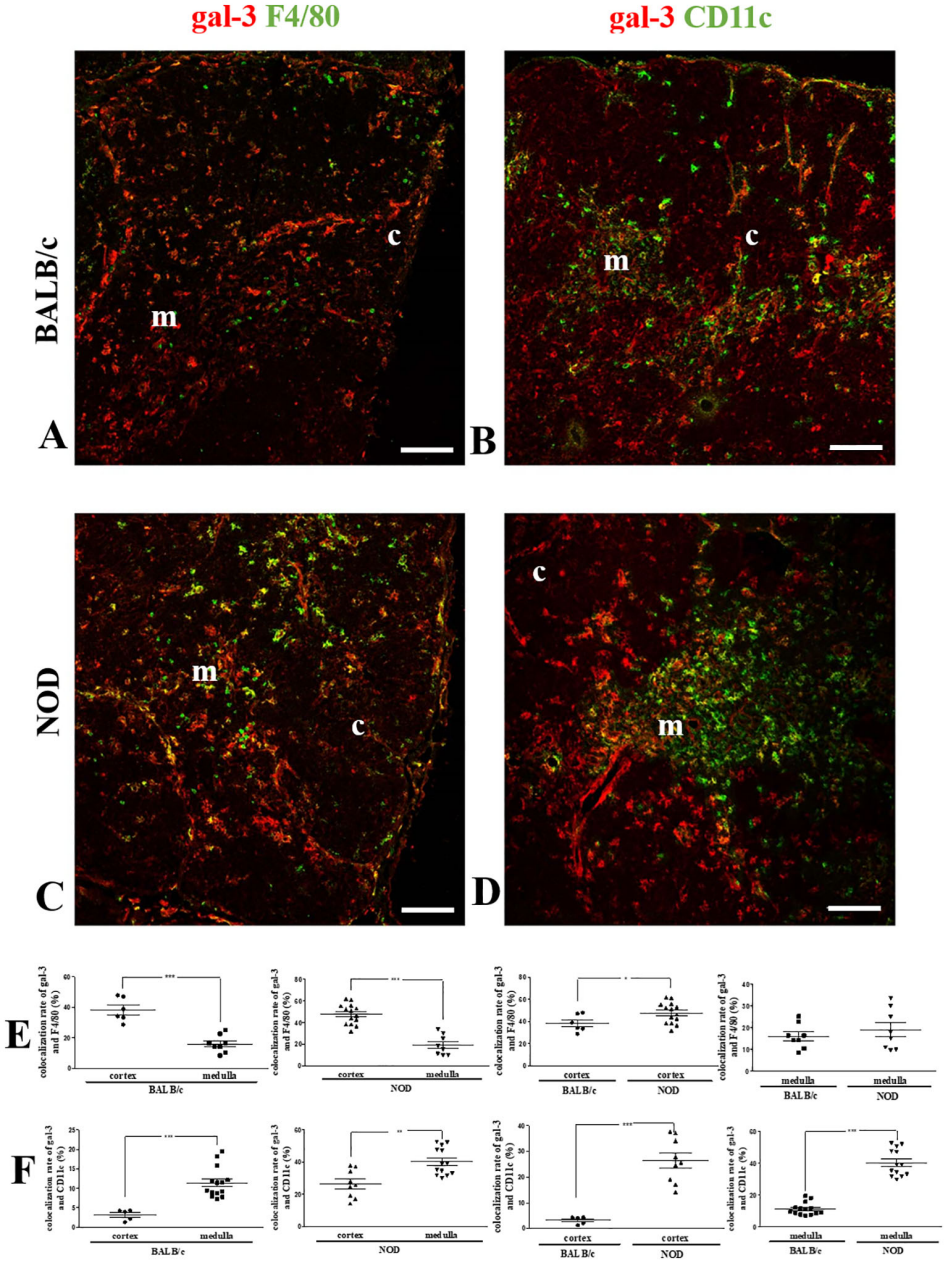
Colocalization of gal-3 with macrophages and dendritic cells in the pre-diabetic NOD mouse thymus. Left panels show double immunostaining for gal-3 (red) and F4/80 (green) in BALB/c **(A)** and pre-diabetic NOD **(C)** mouse thymus sections. Graphs show the colocalization rates of gal-3 and F4/80 **(E)** in the cortical and medullary regions. Right panels show double immunostaining for gal-3 (red) and CD11c (green) in BALB/c **(B)** and pre-diabetic NOD **(D)** mouse thymus sections. Graphs show the colocalization rates of gal-3 and CD11c **(F)** in the cortical and medullary regions. Six to fifteen photomicrographs per group were analyzed (n=3 mice/group). c- cortex; m- medulla. Bar: 100 μm. Data were analyzed by unpaired Student’s *t* test and represented as mean± SEM. *p< 0.05; **p< 0.01; ***p< 0.001.

Next, we double-stained thymus sections with anti-F4/80 and anti-gal-3 antibodies (**Figure 2**). We noticed that NOD mouse thymuses had higher numbers of F4/80^+^ macrophages (**Figure 2C**) than the corresponding BALB/c controls (**Figure 2A**). Interestingly, many BALB/c and NOD cortically located macrophages were also gal-3-positive (**Figures 2A, C**), although most medullary macrophages were negative for the lectin in the thymuses of both mouse strains (**Figures 2A, C**). Quantitative colocalization analysis of gal-3 and F4/80 revealed a statistically significant increase in the colocalization rate in the thymic cortex of BALB/c and NOD, as compared with the respective medullary regions. Yet, NOD cortical (but not medullary) macrophages presented higher gal-3 colocalization than BALB/c controls (**Figure 2E**).

CD11c-positive DCs also expressed gal-3 in both BALB/c and NOD mouse thymuses, with an increased colocalization of these two molecules in the medulla as compared to the cortex in both BALB/c (**Figure 2C**) and NOD mouse strains (**Figure 2D**). Quantitative colocalization analysis revealed that both cortical and medullary DCs in the NOD thymus, express more gal-3, when compared to respective BALB/c controls (**Figure 2F**).

### Intrathymic co-localization of gal-3 with extracellular matrix molecules

**Figure 3:**
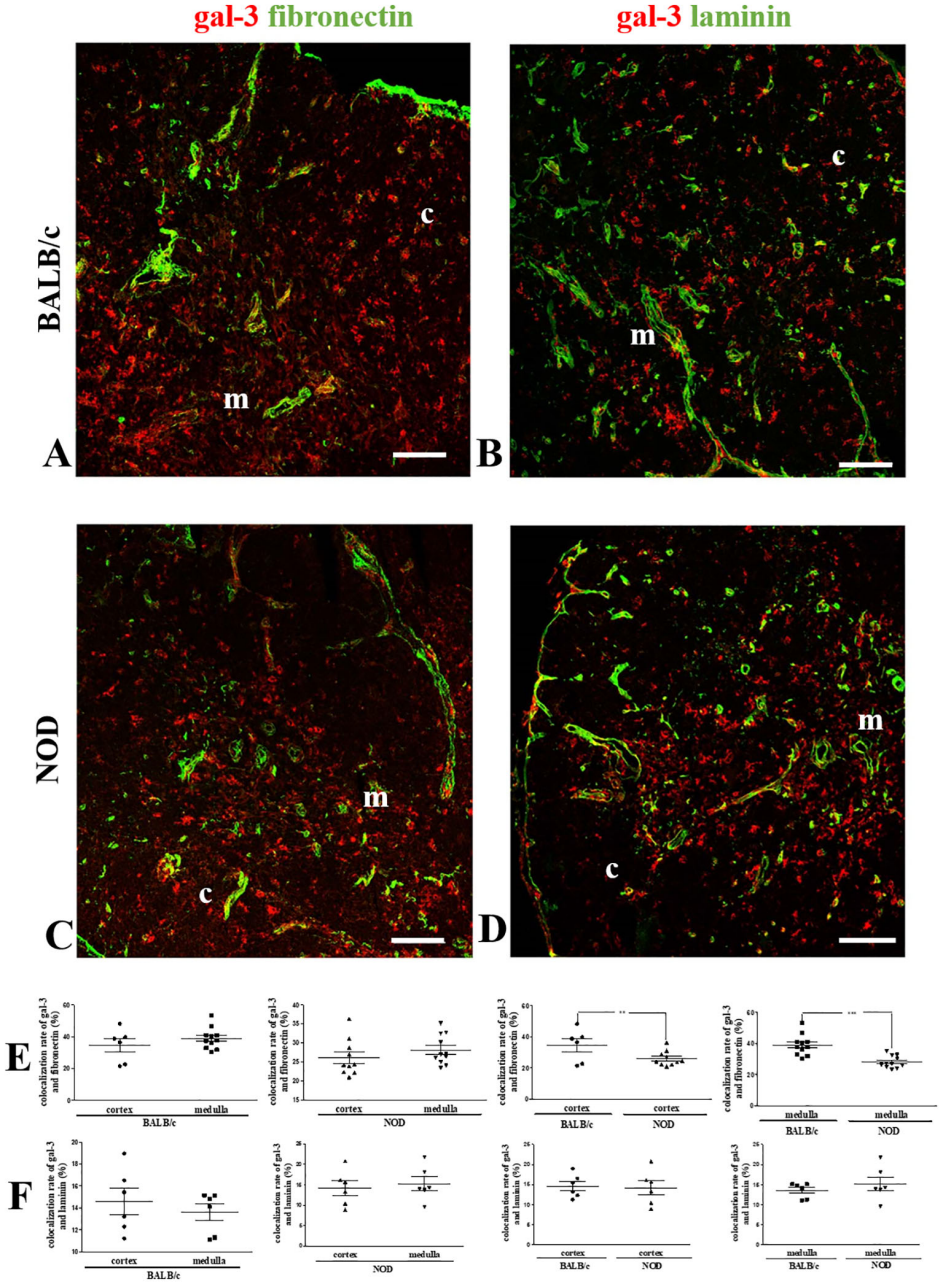
Colocalization of gal-3 and fibronectin in the pre-diabetic NOD mouse thymus. Left panels show double immunostaining for gal-3 (red) and fibronectin (green) in BALB/c **(A)** and pre-diabetic NOD **(C)** mouse thymus sections. Graphs show the colocalization rates of gal-3 and fibronectin in the cortical and medullary regions **(E)**. Right panels show double immunostaining for gal-3 (red) and laminin (green) in BALB/c **(B)** and pre-diabetic NOD **(D)** mouse thymus sections. Graphs show the colocalization rates of gal-3 and laminin in the cortical and medullary regions **(F)**. Six to fifteen photomicrographs per group were analyzed (n=3 mice/group). c- cortex; m- medulla. Bar: 100 μm. Data were analyzed by unpaired Student’s *t* test and represented as mean± SEM. **p< 0.01; ***p< 0.001.

Since gal-3 is secreted by thymic microenvironmental cells and interacts with ECM glycoproteins such as fibronectin and laminin, we also evaluated the colocalization of gal-3 with these molecules (**Figure 3**). No differences in gal-3/fibronectin colocalization rates were observed between the cortex and medullary regions of BALB/c or NOD mouse thymuses (**Figure 3E**). However, a significant decrease in colocalization was observed in the NOD mouse thymus, when compared to BALB/c controls (**Figure 3E**).

Laminin was found mainly in the medulla of both BALB/c and NOD thymuses (42, 43). Like fibronectin, no differences between the thymic regions were observed in both mouse strains when the colocalization analysis of gal-3 and laminin was performed (**Figure 3F**). Conversely, no decrease was observed when the cortex and the medulla of NOD mouse thymus were compared to the respective regions of BALB/c controls (**Figure 3F**).

### Expression of gal-3 and ECM molecules within the intrathymic giant perivascular spaces of NOD mice

**Figure 4:**
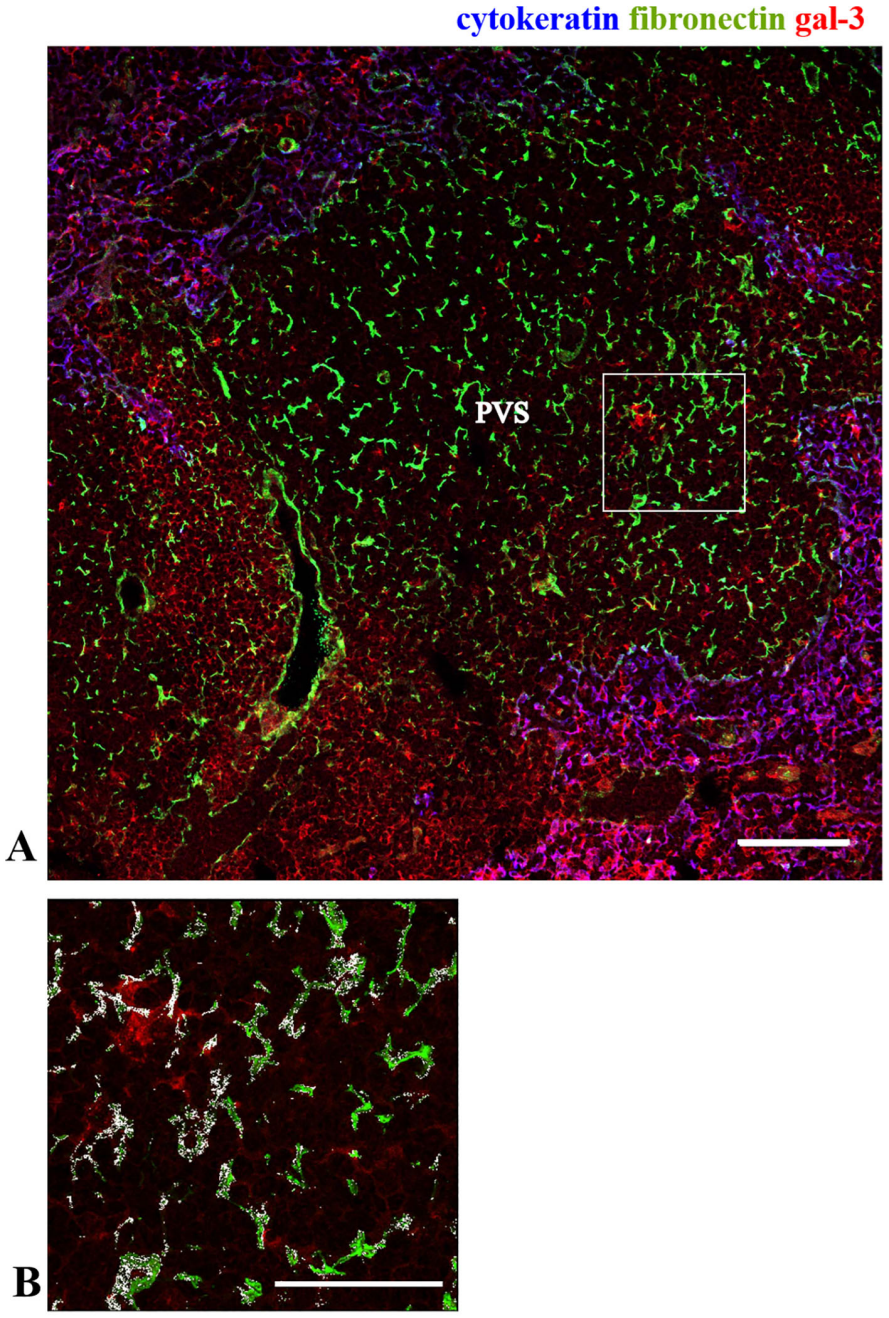
Expression of gal-3 within the giant PVS in the pre-diabetic NOD mouse thymus. Immunostaining for gal-3 (red), cytokeratin (blue) and fibronectin (green) in pre-diabetic NOD mouse thymus. Panel **(A)** shows part of one giant PVS, identified by the lack of TEC, ascertained by the negative staining for cytokeratin. Bar: 100 μm. Panel **(B)** shows a higher magnification of the square marked in the panel **(A)**, with white areas high-lightening the colocalization of gal-3 and fibronectin (n=3 NOD mice). PVS, perivascular space. Bar: 50 μm.

As previously described, one striking thymic alteration in the NOD mouse thymus is the presence of giant PVS, where mature thymocytes are progressively accumulated (35, 36, 38). To define the giant PVS, we performed immunolabeling of thymus sections with anti-cytokeratin, and we analyzed the expression of gal-3 within these structures (negative for cytokeratin). We found a heterogeneous distribution of gal-3 inside the giant PVS (**Figures 4A, B**). We observed an accumulation of fibronectin fibrils within these spaces, with gal-3 being colocalized with some of them (**Figure 4B**). A similar pattern was seen for the colocalization of gal-3 and laminin (**Supplementary Figure 1**).

### Colocalization of Gal-3 with leukocytes within the giant PVS in NOD mouse thymus

**Figure 5:**
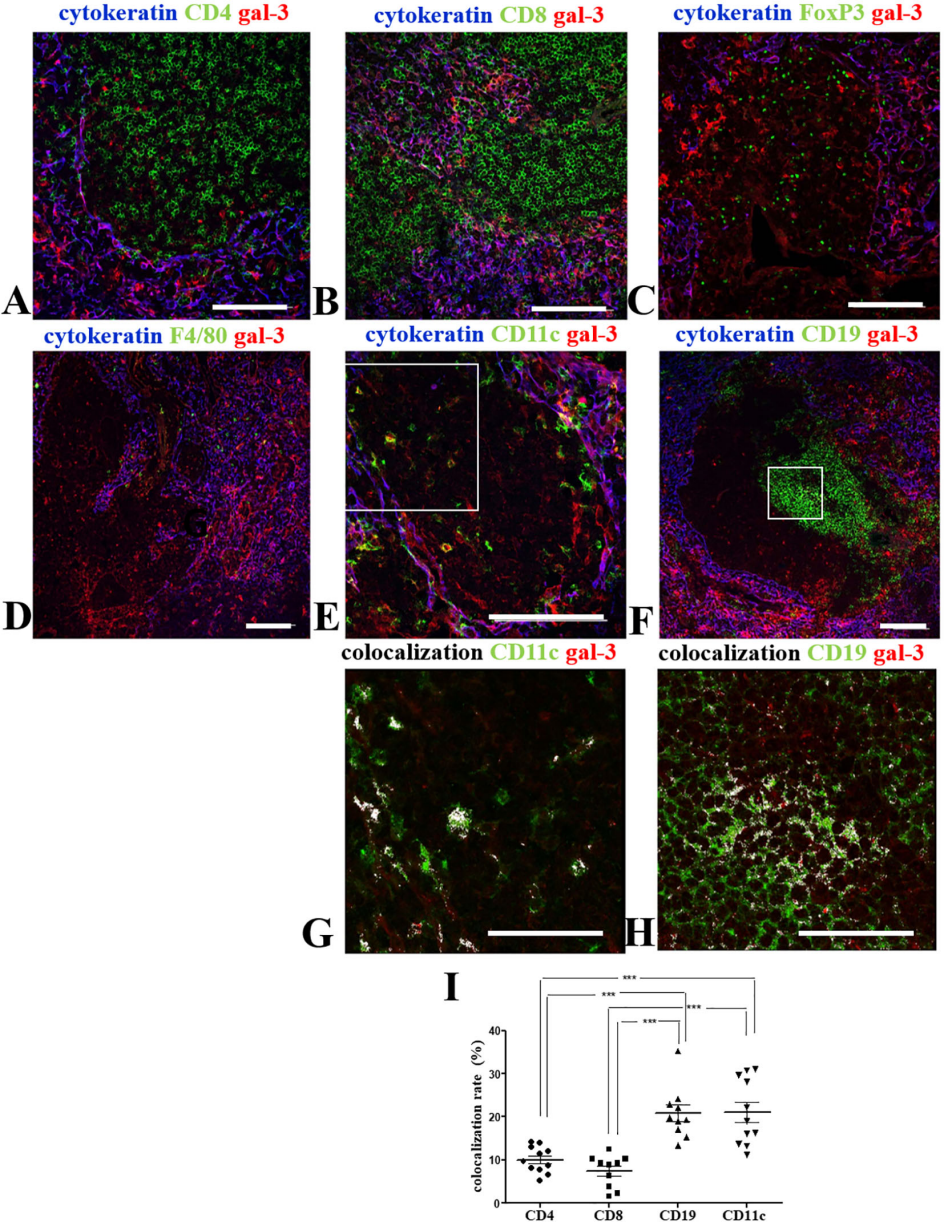
Colocalization of gal-3 with leukocytes within giant PVS of the pre-diabetic NOD mouse thymus. Panels show the triple-labelling of gal-3 (red), cytokeratin (blue) and CD4 **(A)**, CD8 **(B)**, FoxP3 **(C)**, F4/80 **(D)**, CD11c **(E)** or CD19 **(F)** (green) within the giant PVS of pre-diabetic NOD mouse thymus sections (n=3). Bar: 100 μm. Lower panels show higher magnification of the squares, with colocalization for CD11c/gal-3 **(G)** or CD19/gal-3 **(H)** being high-lighted in white. Bar: 50 μm. **(I)** Graph shows the colocalization rates of gal-3 with CD4, CD8, CD19 or CD11c in the giant PVS. Eleven to thirteen photomicrographs per group were analyzed (n=3 NOD mice). Data were analyzed by one-way ANOVA, followed by *post hoc* Tukey’s test and represented as mean± SEM. ***p< 0,001.

Since mature thymocytes are retained within the giant PVS, we investigated the presence of gal-3 labeling, in these cells. For that, NOD mouse thymus sections were labeled for gal-3, cytokeratin, and CD4 (**Figure 5A**), CD8 (**Figure 5B**) or FoxP3 (**Figure 5C**). CD4^+^ thymocytes intermeshed with gal-3 were found inside these structures (**Figure 5A**), as well as a punctuate gal-3 labeling around CD8^+^ and FoxP3^+^ cells within the giant PVS (**Figures 5B, C**).

Differently, we did not observe any F4/80^+^ macrophages in most PVS analyzed, except for the few gal-3-negative-macrophages located near the border of some PVS (**Figure 5D**). Conversely, we did detect gal-3 bearing DCs in giant PVS (**Figures 5E, G**).

Clusters of B cells were described in the giant PVS of NOD mouse thymus (35). Interestingly, we observed a clear colocalization of gal-3 with CD19^+^ cells in intra-PVS clusters of B cells (**Figures 5F, H**). Colocalization rates of gal-3 with CD4, CD8, CD19 or CD11c in the giant PVS were compared (**Figure 5I**). CD11c and CD19 colocalization with gal-3 was clearly seen (**Figure 5I**). Although no colocalization between FoxP3 (nuclear staining) and gal-3 was observed, FoxP3^+^ cells are positioned intermingled in a gal-3-rich environment (**Supplementary Figure 2**).

Together, these data suggest a close relationship of leukocytes with gal-3 within the giant PVS observed in pre-diabetic NOD mouse thymuses.

### Gal-3-induced thymocyte migration is impaired in the NOD thymus

**Figure 6:**
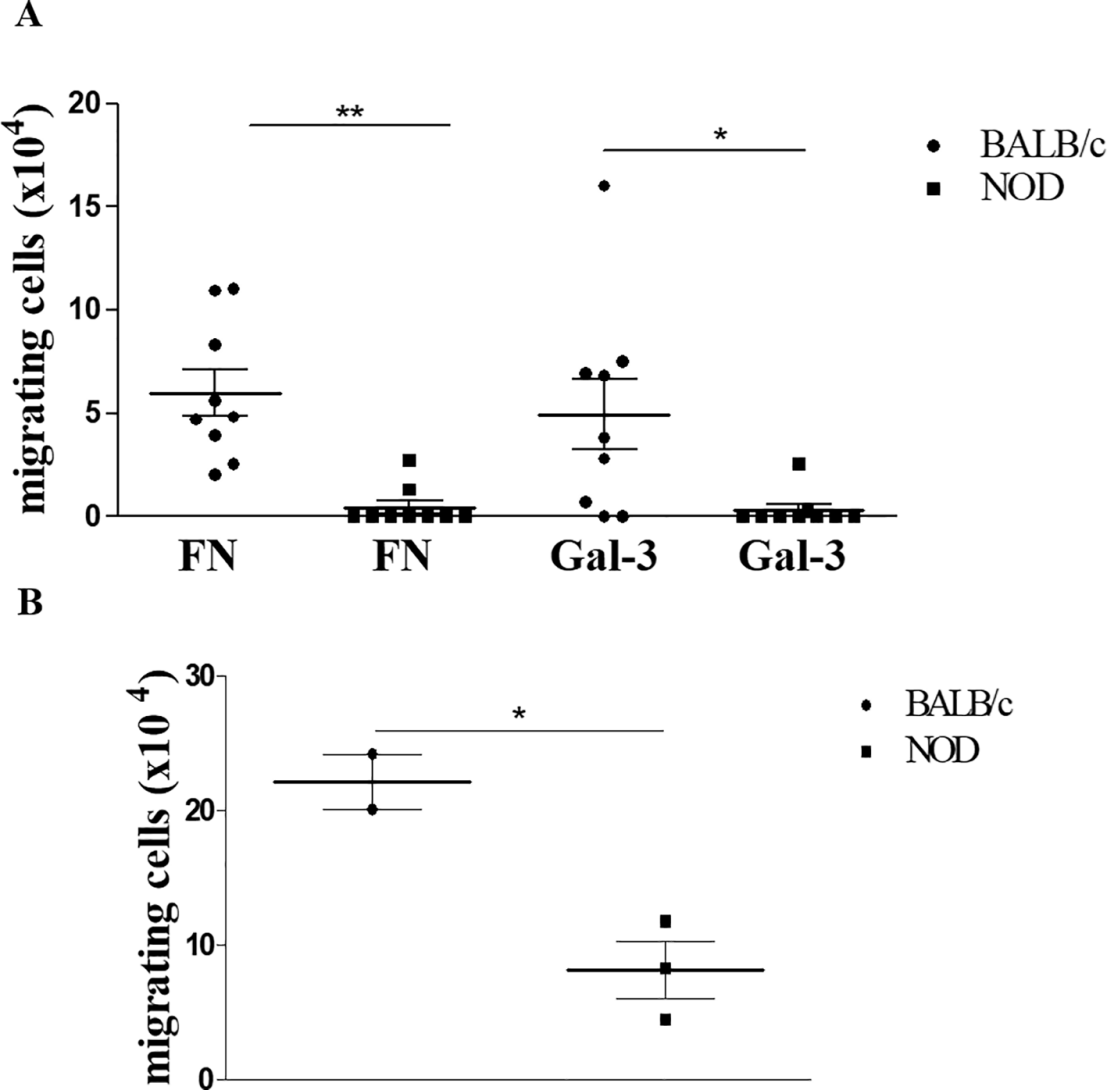
*Ex-vivo* gal-3-driven thymocyte migration is impaired in pre-diabetic NOD mice. Panel **(A)** shows the migratory response of thymocytes from BALB/c and NOD mice upon fibronectin or gal-3 stimuli, showing that NOD thymocyte migration is largely reduced under any stimuli tested. Data represent three individual experiments with 3 animals per group, showing the specific migration, after subtracting the values obtained from non-specific migration recorded under BSA treatment. Data were analyzed by one-way ANOVA, followed by *post hoc* Tukey’s test, and expressed as mean± SEM. Similar results were seen in panel **(B)**, which describes reverse transendothelial cell migration mimicking thymocyte egress from the thymus. Data were analyzed by unpaired Student’s *t* test and expressed as mean± SEM, with 3 animals per group. *p<0.05; **p< 0.01.

Considering our previous data showing that gal-3 is capable of interfering with the interaction of thymocytes with thymic microenvironmental cells modulating their migration and adhesive properties (19), we evaluated the possible influence of gal-3 on NOD thymocyte migration. Firstly, we confirmed that thymocytes from NOD mice have a significant decrease in the migratory response towards fibronectin when compared to control BALB/c derived thymocytes. Importantly, a decrease in the migratory response of NOD thymocytes was also seen in response to gal-3 itself (**Figure 6A**).

Given the previous data showing that mature thymocytes from NOD mice are accumulated within giant PVS that occupy a large part of the medullary thymic region (35), and considering that NOD thymocytes have a deficient migratory response in response to gal-3, we decided to evaluate the role of this lectin in thymocyte egress using a reverse transendothelial cell migration assay. We initially assessed gal-3 expression in the mouse thymic endothelial line tEnd.1, demonstrating that these cells produce gal-3 (**Supplementary Figure 3**). To carry out the transendothelial migration assay, we cultured the thymic endothelial cells in a transwell membrane in order to form a monolayer which the thymocytes would be able to cross, mimicking their exit through the blood-thymus barrier, as previously described (38). We observed that NOD thymocytes exhibited deficient reverse transendothelial migration compared to BALB/c thymocytes (**Figure 6B**). Although preliminary, our data suggest that gal-3 is involved in the migration disturbs observed in the thymus of pre-diabetic NOD mice.

## Discussion

**Figure 7:**
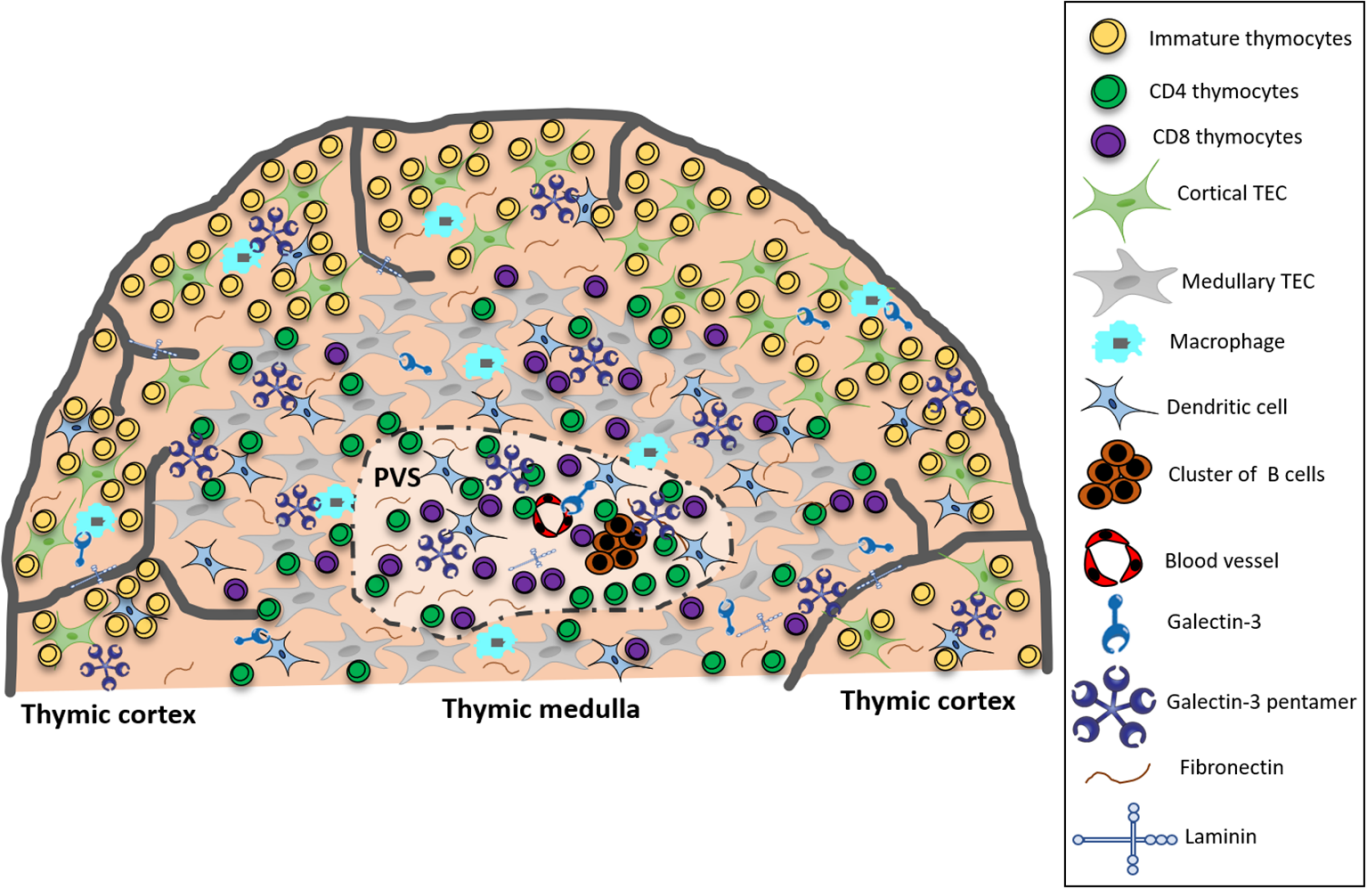
Summarizing scheme showing that in the pre-diabetic NOD mouse thymus, gal-3 in the cortex colocalizes predominantly with macrophages, whereas in the medulla, colocalization is primarily with TECs and DCs. Within the giant PVS, gal-3 colocalizes with clusters of B cells, DCs, fibronectin and laminin molecules, and is intermingled with T cells. Taken together, these data suggest that gal-3 is involved in intrathymic disorders observed in NOD mice. PVS, giant perivascular space; TEC, thymic epithelial cell.

In the present study, we characterized gal-3 expression in thymic microenvironmental cells and the colocalization of this lectin with ECM molecules in the pre-diabetic NOD mouse thymus. Our colocalization analyses of gal-3 with specific cell markers (cytokeratin, F4/80 and CD11c) by immunofluorescence strongly suggested that gal-3 is produced by thymic microenvironmental cells. However, we cannot discard the possibility that the intracellular gal-3 detected by this technique could represent gal-3 endocyted by microenvironmental cells. Also, the accumulation of secreted gal-3 in the microenvironment outside the cells, bound to ECM glycoproteins or even bound to membrane glycosylated antigens is to consider. Additionally, we showed that gal-3-driven cell migration is altered in NOD thymocytes. Our results are summarized in **Figure 7**.

We have previously reported a partial colocalization between gal-3 and cytokeratin in BALB/c mouse thymus, suggesting that gal-3 is produced by TEC. Further we detected the presence of gal-3 in the surface and cytoplasm of the thymic epithelial cell line IT-76M1, by immunofluorescence and flow cytometry, as well as its secretion to the culture supernatant (19). Based on these results, we first evaluated the expression of gal-3 in TEC and observed an increase of colocalization between gal-3 and cytokeratin in both cortical and medullary regions of NOD thymus, when compared to the corresponding thymic regions in BALB/c mice. Since no increase of cytokeratin-positive epithelial cells was reported in the NOD thymus (38), it seems likely that NOD TEC actually presents an enhancement of gal-3 expression. In fact, we observed an increase in the proportion of gal-3 producing cells with age in both thymic cortical and medullary regions in NOD mice as compared to BALB/c age-matched controls (**Supplementary Figure 4**).

We also studied gal-3 expression in non-epithelial (cytokeratin-negative) gal-3-positive microenvironmental cells in the NOD mouse thymus, in both macrophages (F4/80 positive) and dendritic cells (ascertained by CD11c labeling). Of note, many cortical macrophages were phenotypically F4/80^+^gal-3^+^, while medullary macrophages are rather F4/80^+^gal-3^-^. An immunohistochemical study identified three types of macrophages, namely dendritic, round, and flat-shaped in the mouse thymus. Dendritic macrophages are localized throughout the thymus. Most of the round macrophages are distributed in the cortico-medullary junction and medulla. In contrast to our results, these authors showed that round medullary macrophages are phenotypically F4/80^-^gal-3^+^ (44). Since other cells express gal-3 in the thymus, we consider that this phenotype does not assure the exclusive identification of these cells as macrophages. The morphological and phenotypical heterogeneity of thymic macrophages suggests that they would play distinct roles in thymic regions where they are located. By electron microscopy studies, dendritic macrophages containing phagosomes with ingested thymocytes were observed, indicating that these cells are responsible for phagocytosis of apoptotic thymocytes (45). Regarding the round macrophages, it is possible that they are involved with the final stage of thymocyte differentiation, based on their location. In addition, round macrophages could represent an immature population of macrophages that recently entered the thymus via blood vessels running through the septa to the medulla and/or cortico-medullary junction (44, 46, 47).

Also, we showed that the co-localization of gal-3 in NOD thymic DCs was increased as compared to controls. Basically, DC are divided in two subsets: plasmacytoid and conventional DC (48). Herein, it was not possible to distinguish which subset of DC is increased by immunofluorescence, because CD11c is expressed in both DC subsets. The increase of DC number was also detected in old BWF1 (NZB x ZNW) mice, which spontaneously develop systemic lupus erythematosus like humans. Moreover, thymic CD11b^+^CD11c^+^ DC were identified as the main source of BLC/CXCL13, important chemokine for B1 cells chemoattraction via the CXCR5 receptor (49). According to these authors, an abnormal presence of DC in the thymus could be related to positive selection of autoreactive T cells. The increased number of CD4 T cells and decrease of double-positive cells are important findings that fit this hypothesis. Nevertheless, it does not seem to apply to NOD mice, due to their apparently normal T cell differentiation (37). As the presence of B cell clusters is one of the striking alterations in NOD mouse thymus, it would be interesting to examine CXCL13 expression by thymic DC to associate to B cell recruitment to the thymus.

We observed a decrease in colocalization between gal-3 and fibronectin in the NOD thymic parenchyma when compared to BALB/c thymus, despite the increase of fibronectin deposition in the thymus of these animals that we previously reported (38). Concerning laminin, we did not observe any difference in the colocalization with gal-3. In conjunction, these results suggest that NOD thymic microenvironmental cells possibly retain gal-3 or have a decrease in secretion of this protein. The evaluation of the presence of gal-3 in the microenvironmental cell supernatant would be a suitable experiment to test this hypothesis. Since thymocyte migration is guided by combined stimuli of ECM proteins and soluble molecules such as gal-3 (21, 38), one might wonder that the decrease of colocalization between these proteins alter NOD thymocyte migration.

Importantly, gal-3 was also seen in the giant PVS, one of the most important morphological alterations described in the NOD mouse thymus. Curiously, the enlargement of these structures was observed in other autoimmune diseases, such as in the thymus of myasthenia gravis patients (7) and BWF1 mice (50). Besides the cellular staining in both TEC, macrophages and DCs, we showed that gal-3 is deposited on the ECM network that intermingles with the cells within PVS. Moreover, we noticed an intense expression of gal-3 in blood vessels within PVS, which is in the same line as the fact that this lectin was also observed in the thymic endothelial cell line studied herein. The expression of this lectin was also observed in the endothelial cell line HUVEC as well as in the endothelium of some organs, including kidney, placenta and colon (51). In the present study, gal-3 produced by the endothelial cells of giant PVS might modulate thymocyte emigration.

Considering that mature CD4^+^SP and CD8^+^SP T cells, regulatory T cells, and B lymphocytes are progressively retained in the giant PVS (35, 38), we analyzed if gal-3 was also colocalized with these cell types. Of note, a small part of B cell clusters present gal-3 bound to their surface. On the other hand, CD4, CD8 and regulatory T cells were found intermingled with gal-3, probably produced by other cells or deposited on ECM components. The accumulation of B cells in the PVS was also described in the NOD mouse thymus (52). In addition to T cells, we previously reported that B cells exhibit a defect in VLA-5 expression that could justify the arrest of these cells in these structures (37, 38). One possible consequence of their retention might be the production of natural autoantibodies, important biomarkers of type 1 diabetes. In this context, we have previously detected the presence of deposits of immunoglobulins on TEC and fibronectin fibers, revealed with fluorescent-labeled anti-mouse Ig sera (35). The giant PVS of old BWF1 mouse thymus is also infiltrated by B cells (50). In these animals, B cell recruitment occurs during the development of lupus nephritis and is associated with abnormal expression of CXCL13 and adhesion molecules, such as PNAd, ICAM and VCAM-1 in the blood vessels of PVS. Furthermore, it is possible that B cells play a role in the activation of autoreactive T cells that express TCR of low avidity and escape to the periphery. The origin of thymic B cells in NOD mice was not yet unraveled. On one hand, we can suppose that B cells are circulating cells from the periphery that enter the thymus. On the other hand, thymic B cells could originate from intrathymic precursors. A recent study demonstrated that the majority of thymic B cells of a normal mouse develop inside the organ and that peripheral recirculation has a small contribution to thymic B cell population (4).

To the best of our knowledge, this is the first report of DCs within giant PVS. As seen in the parenchyma, gal-3 colocalizes with these cells. In addition to T cells and B cells, there is a low expression of VLA-5 receptor in DC that could again justify their retention in PVS (38).

We have previously demonstrated that gal-3 modulates thymocyte adhesion/de-adhesion in physiological and pathological conditions (19, 21), so it is possible that gal-3 accumulation in the NOD mouse thymus microenvironment is related to the increase of adhesion between thymocytes and the thymic stroma, impairing thymocyte migration and exit from this organ. This effect can be explained by the fact that gal-3 is able to form oligomers at high protein concentrations through its N-terminal portion. In this manner, gal-3 could induce adhesion between different cells or between cells and ECM (14, 15, 53).

Another possible consequence of gal-3 accumulation in thymic microenvironmental cells is the increase of its secretion to the extracellular space that could contribute to the endocytosis of VLA-5 fibronectin receptor in NOD thymocytes. Gal-3-mediated-endocytosis of β1 integrins in breast cancer cell lines reinforces this idea (54). Accordingly, we conjecture that extracellular gal-3 enhancement could contribute to VLA-5 deficiency described by our group in the thymus of NOD mice (37).

Considering that gal-3 mediates the migration of different cell types including thymocytes (21, 55–57), and since this lectin can bind to the fibronectin receptor VLA-5 (16–18), whose membrane expression is impaired in NOD thymocytes (37, 38), we evaluated the effect of gal-3 on NOD thymocyte migration. NOD thymocyte migration *ex vivo* was largely impaired, to a similar extent, under fixed concentrations of fibronectin and gal-3 stimuli. Although hypothetical, one potential reason is that gal-3 binding to VLA-5 is necessary for generating a migratory response of developing thymocytes, stimulated by gal-3 alone (18).

A further point deserving to be addressed is that deficient migration under gal-3 in NOD thymocytes was not reversed in the presence of fibronectin (data not shown), suggesting that gal-3 induces thymocyte migration via triggering VLA-5, as recently demonstrated in other cell types (18). The additive synergism of gal-3 with fibronectin in migration has been reported in tumor mammary epithelial cells (58). These authors showed that the interaction of gal-3 with VLA-5 leads to the activation of focal adhesion kinase (FAK) and phosphatidylinositolphosphate (PI3K), important events for fibronectin fibrillogenesis and cell motility. Furthermore, this effect is carbohydrate-dependent, as it is abrogated in the presence of lactose, a competitive inhibitor of gal-3. Peptides containing the RGD sequence (arginine-glycine-aspartate) are known to block the interaction of VLA-5 and fibronectin, by competing with the binding of this receptor to its specific fibronectin binding site (59), and consequently inhibit cell migration. However, when gal-3 was added to the assay in the presence of RGD-containing peptides, cell migration was increased, suggesting that the interaction of fibronectin and VLA-5 mediated by gal-3 is RGD-independent but N-dependent. -glycans present in the molecule of this β-1 integrin (58).

In the last years, we established the concept of multivector cell migration, which proposes that thymocyte migration is a complex process resulting from multiple and simultaneous interactions of extracellular matrix molecules, gal-3, chemokines, and hormones with their respective receptors (38, 60). This concept has been extensively explored in pathological situations, in which this multivector migration is altered. In the present study, we obtained strong evidence that the individual vector directed by gal-3 is reduced in the multivector migration of NOD thymus. This is the description of a second vector apparently altered because of the VLA-5 expression defect. However, a dissection of the signaling pathways involved in VLA-5 deficiency is necessary for a better understanding of the role of this integrin in the pathogenesis of autoimmune diabetes.

Lastly, to investigate whether the deficiency in the migratory response to gal-3 would contribute to the retention of mature thymocytes within the giant PVS in NOD mouse thymus, we used an *ex vivo* model of transendothelial migration which revealed significantly decreased migratory response of NOD thymocytes in the presence of gal-3. Again, it is possible that gal-3 uses VLA-5 to induce transendothelial cell migration. Complementary assays using blocking antibodies or siRNA are needed to confirm this hypothesis and if thymocyte transendothelial migration is dependent on gal-3, which will certainly contribute to the understanding of the retention of mature thymocytes in NOD mouse thymus. In any case, altogether, our data unravel an altered expression of gal-3 in the NOD mouse thymus, with likely consequences upon thymocyte migration.

## Data Availability

The raw data supporting the conclusions of this article will be made available by the authors, without undue reservation.
