# Supplementary material for: Altered galectin-3 distribution and migratory function in the pre-diabetic non-obese diabetic mouse thymus
Source: Front Endocrinol (Lausanne). 2024 Oct 17;15:1200935. doi: 10.3389/fendo.2024.1200935 (PMC11524864; doi:10.3389/fendo.2024.1200935)
Supplement: Supplementary file 1 [file DataSheet1.pdf]

Supplementary Material

Supplementary Figure 1

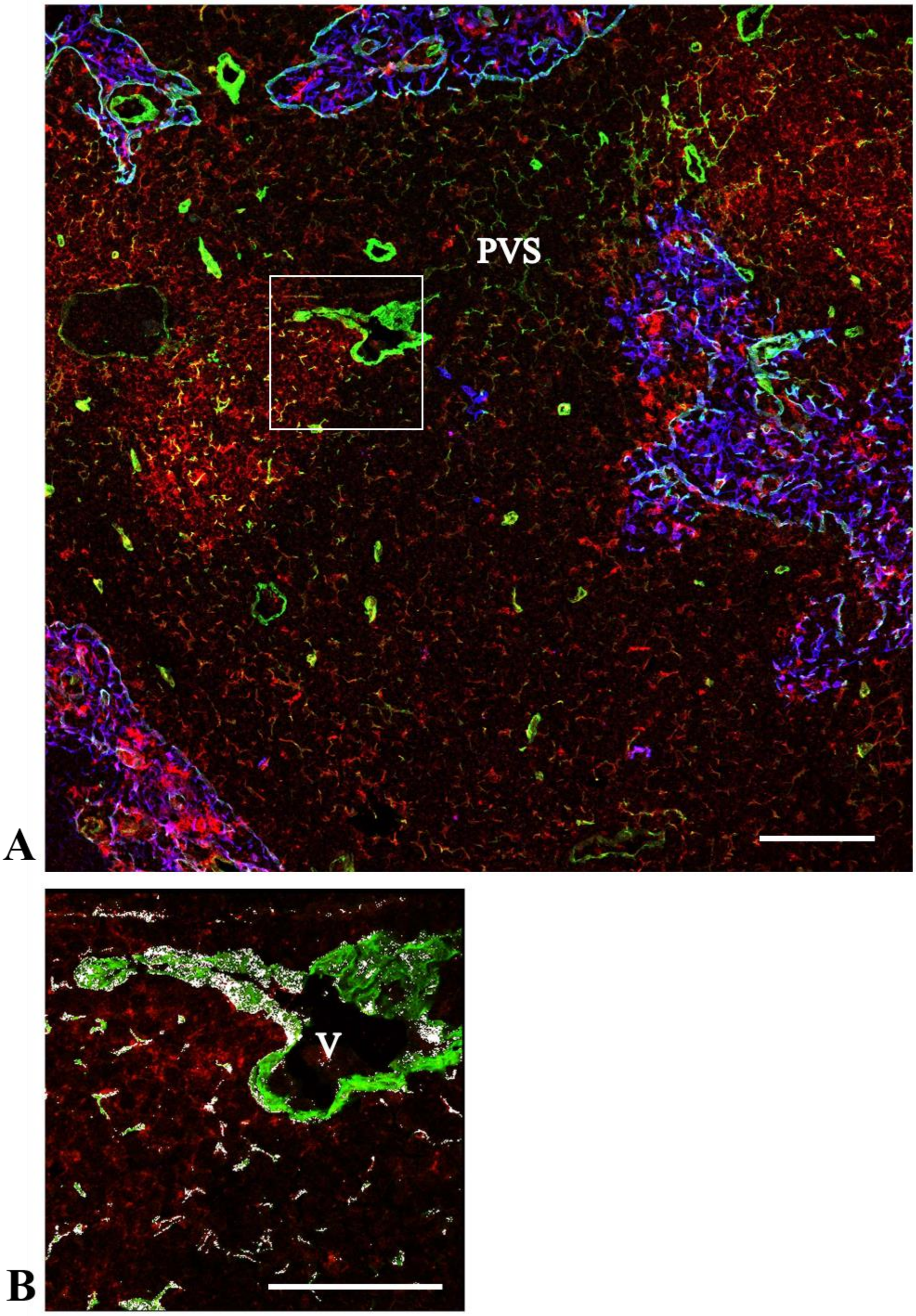

Supplementary figure 1. Colocalization of gal-3 with laminin in the giant PVS of a NOD mouse thymus. Immunostaining for gal-3 (red), cytokeratin (blue) and laminin (green) in pre-diabetic NOD mouse thymus. Panel (**A**) shows part of one giant PVS, identified by the lack of TEC, ascertained by the negative staining for cytokeratin. Bar:

100 μm. Panel (**B**) shows a higher magnification of the square marked in the upper panel. Laminin is expressed in the giant PVS and lining blood vessels, with white areas high-lightening the colocalization of gal-3 and laminin. PVS- perivascular space; V- blood vessel. Bar: 50 μm.

Supplementary Figure 2

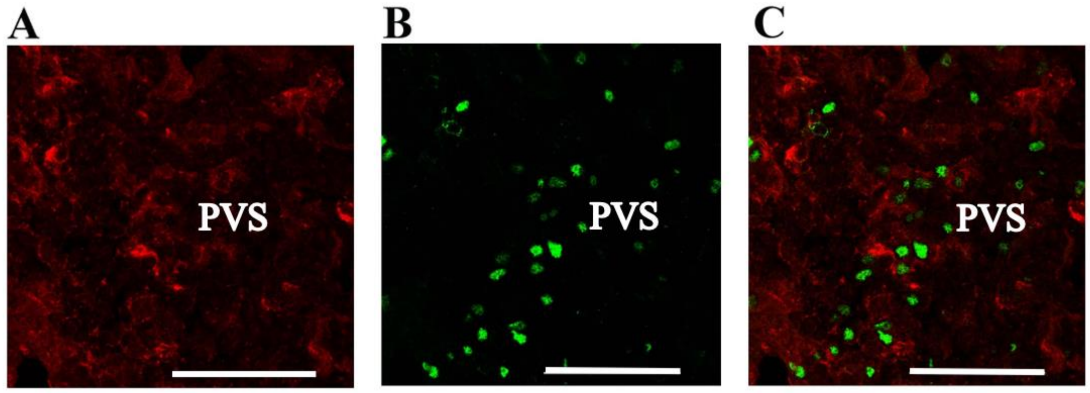

Supplementary figure 2. FoxP3$^+$ cells in the giant PVS are intermeshed with a gal-3-rich environment. Deposits of gal-3 are detected around regulatory (FoxP3+) T cells within the giant PVS of a pre-diabetic NOD mouse thymus section. (**A**) Single staining for gal-3 in PVS. (**B**) Single staining for FoxP3. **(C)** Merge of gal-3 and FoxP3. PVS- perivascular space. Bar: 50 μm.

Supplementary Figure 3

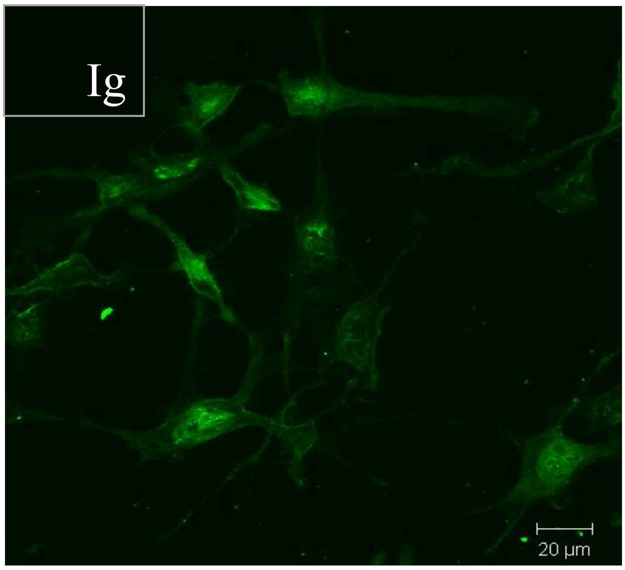

Supplementary figure 3. Expression of gal-3 in thymic endothelial cells. Immunostaining for gal-3 in the t.End.1 mouse thymic endothelial cell line shows its presence on the cell surface, cytoplasm, and nucleus. Insert represents a negative control (with no labeling) when anti-gal-3 antibody was replaced by an unrelated Ig (Ig). Magnification can be seen in the bar, with 20 μm.

Supplementary Figure 4

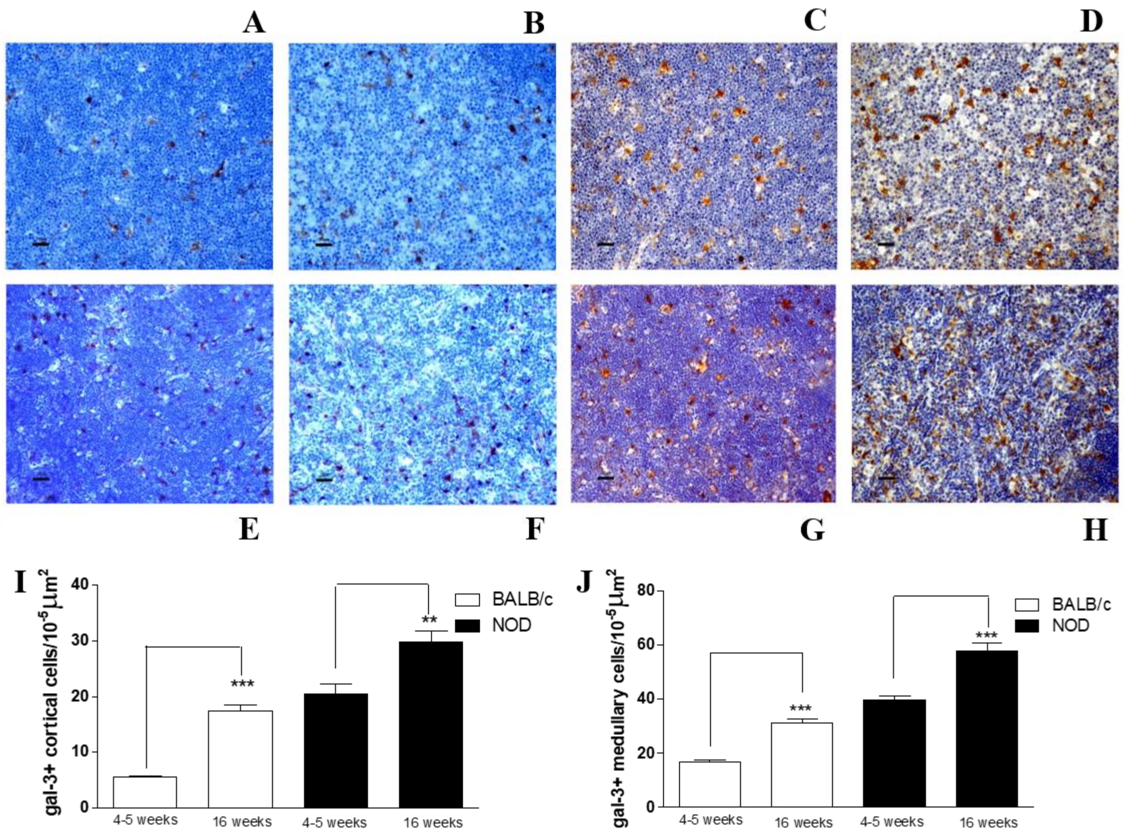

Supplementary figure 4. Immunohistochemical localization of gal-3 in BALB/c and pre-diabetic NOD mouse thymuses aged 4-5 weeks and 16 weeks. (**A**) and (**B**) correspond respectively to cortical and medullary regions from to 4-5 weeks BALB/c mouse thymuses. (**C**) and (**D**) correspond respectively to cortical and medullary regions from to 4-5 weeks NOD mouse thymuses. (**E**) and (**F**) correspond respectively to cortical and medullary regions from to 16 weeks BALB/c mouse thymuses. (**G**) and (**H**) correspond respectively to cortical and medullary regions from to 16 weeks NOD mouse thymuses. Counterstaining with hematoxylin. Scale bar: 25 µm. Quantitative analysis of selected cortical (**I**) and medullary regions (**J**) of BALB/c and pre-diabetic NOD mouse thymuses. Quantitative analysis was performed by dividing the number of gal-3 positive cells per area in μm^2 (n=3 thymuses/group). Data were analyzed by unpaired Student's *t* test and expressed as mean ± SE. **$p<0.01$; *** $p < 0.001$.
